# ‘Health Camp’ model: a unique approach for child vaccination in non-state armed actor controlled, inaccessible geographies in Somalia

**DOI:** 10.1080/16549716.2024.2391598

**Published:** 2024-08-23

**Authors:** Mohamed Hussein Kahow, Salad Ahmed Halane, Asma Ali, Rashed Shah

**Affiliations:** a Health Technical Specialist – Immunization, Save the Children International, Mogadishu, Somalia; b Health Systems Strengthening, Ministry of Health – Galmudug, Dusmareb, Somalia; c Global Development, Bill and Melinda Gates Foundation, Mogadishu, Somalia; d Department of Global Health, Save the Children US, Washington, DC, USA

**Keywords:** Health Camp, immunization, Somalia, inaccessible area, zero-dose children

## Abstract

Decades of conflict, political instability, and limited infrastructure left Somalia facing significant challenges to offer consistent and equitable health services, especially for child vaccination. Recent data reveals alarming vaccination gaps, with 60% of children receiving no vaccinations, and only 11% completing required vaccines. Despite global support, an estimated 1.15 million children remain unvaccinated, half of them reside in inaccessible areas controlled by non-state armed actors. In this context, the Far-Reaching Integrated Delivery (FARID) project was initiated since October 2022 across 10 districts of Galmudug and Hirshabelle state in Somalia. Employing the ‘Health Camp’ model, FARID addresses social, structural, and gender barriers, adapting to ever-changing context of inaccessible regions by providing mobile health facilities and outreach health and nutrition services, including child vaccination. This approach effectively reached previously unreached population in Somalia’s most difficult-to-reach areas. Implemented in phases, the project immunized 51,168 children (0–23 months) who had not received any prior vaccinations (23,753 boys and 27,415 girls), screened and treated 14,158 malnourished children (0–59 months) and vaccinated 11,672 pregnant women during March–December 2023. The project’s success hinges on intensive community engagement, local partnerships, innovation in mapping and data management, and delivery of integrated services tailored to population needs. The project underscores the critical role of local community-based organizations and clan elders in reaching inaccessible populations through humanitarian negotiation amidst security challenges. The project has achieved significant milestones aligned with national health strategic plans, including progress towards universal health coverage and improved immunization access in Somalia’s most challenging regions.

## Paper context

**Main findings:**

To improve immunization coverage in areas with access constraints, programs must be integrated, utilize innovation in data systems and mapping and have deep knowledge of humanitarian access negotiation techniques and principles.

**Added knowledge:**

Rights-based approaches including participation, empowerment and accountability are key for a successful immunization program for equitable access.

**Global health impact for policy and action:**

The strengthening of human rights for health through inclusion of populations living under non-state actors’ control in national health policies and legal frameworks is key for equitable access to vaccination to prevent and stop mortalities and morbidities caused by outbreaks and pandemics.

## Background

Following decades of conflict, political instability, and with limited capacity, infrastructure, and management capacity, Somalia is one of the most difficult places in the world to provide consistent and continuous health services. This is underscored by the outcomes of the Expanded Program for Immunization (EPI), as indicated in the most recent Demographic Health Survey data, which reveals that 60% of children received no vaccinations at all, and overall, 11% of children had received all required vaccines [1].

While development partners [including The Bill and Melinda Gates Foundation, Global Alliance for Vaccine and Immunization, World Health Organization (WHO), United Nation Children’s Emergency Fund (UNICEF), World Food Program (WFP), World Vision and Save the Children] are supporting both federal and state level Ministry of Health (MoH) in Somalia for activities to ensure every child’s vaccination in Somalia, an estimated 1.15 M children reported to be consistently missed, by the routine immunization services and Polio campaigns. Half a million of these children live in inaccessible geographies [2].

These inaccessible areas are controlled by non-state armed actors, with minimal or no humanitarian agencies in operation. The population is largely rural and remote, with livelihoods more fragile than those in accessible or government-controlled areas. Their needs are further exacerbated by the global economic recession, the impact of COVID-19, climate change and food price inflation, amidst diminishing oversees remittance and donor funding.

## ‘FARID’ project

Since October 2022, Save the Children in Somalia, with support from the Bill and Melinda Gates Foundation, has been implementing the Far-Reaching Integrated Delivery (FARID) project. The aim of this community-based initiative is to immunize zero dose children and enhance continuous access to quality Immunization within inaccessible geographies across 10 districts in Galmudug and Hirshabelle states in Somalia. These districts include El Dheere, Elgaras, Galcad, Buulo Burte, Beledweyne, Jalalaqsi, Adale, Aden Yabaal, Run-Nirgood and Xarardheere. The FARID project also includes an extensive demand creation and community engagement component to build the trust of the target communities and improve their health seeking behavior. Save the Children in Somalia complements the delivery of health services offered through the ‘Health Camp’ model leveraging its network and resources of ongoing projects and offered mobile outreach health and nutrition services, including child vaccination.

## ‘Health Camp’ model

The ‘Health Camp’ model considers social, structural, and gender barriers prevalent among the populations living in inaccessible areas. These health camps are mobile health facilities, moving around districts to deliver basic health and nutrition packages that focus on maternal health, EPI, and nutrition. The project design was informed by feasibility assessments and is adaptive to changes in the context.

Each health camp is planned for five consecutive days, repeated monthly. This model is delivered through partnership with local organizations able to operate in areas controlled by non-state armed actors. Access to these areas has been facilitated through negotiation with community leaders, clan elders and relevant non-state actors. The design of ‘Health Camp’ model was guided by standard humanitarian principles (Humanity, Independence, Impartiality, and Neutrality) [3]. Also, the project design was informed by feasibility assessments and adaptive to changes in the context.

## How ‘Health Camp’ model operates

In response to varying levels of insecurity within the project targeted 10 districts, FARID project operationalized a rapid and intensive scale-up of immunization services following a phased approach. Two districts (Beledweyne and Xarardheere) were the first pilot districts in March 2023, and the remaining districts were reached progressively through clan-led access negotiation approach. Before initiating health camps, with collaborative support from other consortium partners and other actors (e.g. WHO, MoH). Save the Children gathered contextual information about each district to help design macro-plans for each of these 10 districts. Simultaneously, clan elders mediated access negotiation process which involved three successive roundtable meetings. Upon successful negotiation discussion and securing the green signal for access, community-led microplanning was initiated followed by community guided recruitment process and sourcing of other project inputs. Implementation through local organization, while monitoring and evaluation of the project was done by independent monitor in close collaboration with community elders. The WFP provided logistic support to deliver essential project inputs (medical supplies and cold chain equipment) to the regional warehouses to ease last mile delivery by local partners.

Save the Children engaged two local non-government organizations (NGOs): Development for Humanity and Wardi Relief and Development Initiative (WARDI) to deliver this model by setting up hubs in the main urban centers of the respective districts. The main actor was then linked to these local partner NGOs, who worked with the target community to identify locally available resources to set-up these hubs and implemented the project. These hubs provide a base where mobile health teams stored their supplies, organized their health camp missions, and replenished vaccines and other supplies. All the consortium partners maintained a low-profile, monitoring the security situation and ensuring contingency plans to safeguard project personnel and assets, along with continuing coordination and communication with community leaders. Through effective coordination with the ecosystem of partners, the project team closely monitored emerging issues and proactively mobilized necessary support for effective responses to any urgency.

The collaborative project team also conducted a mapping of the existing health infrastructure in the target districts and establish coordination links and referral pathways to complementary services not offered by the health camps, with emphasis on referral pathways for cases with critical conditions. The team also worked together with Humanitarian Dialogue, the coordinating NGO along with local communities through community health committees to promote access to, and utilization of, the health camps.

## Achievements and challenges

During March–December 2023, ‘Health Camp’ teams identified and immunized 51,168 (23,753 boys and 27,415 girls) zero dose children (0–23 month) with routine immunization, screened and treated 14,158 malnourished children (0–59 months) and vaccinated 11,672 pregnant women and offered other primary health care packages. In mid-Sept 2023, access to all the 10 districts was secured and health camp teams ensured delivery of integrated essential package across all the target districts by building capacity of EPI nurses and assistants, equipping them adequately to provide quality immunization services and by establishing functional Community Health Committees to support efficient implementation and monitoring of services offered through health Camps. Occasionally, the teams had to suspend services owing to renewed threats of insecurity or active conflict between government forces and Al-Shabaab. In June 2023, an immunization volunteer team, consisting of four local NGO staff, was ambushed by Al-Shabaab in Qarsooni villages, Beledweyne district, Hiran region, Hirshabelle state. These team members were held hostage for 2 weeks, impacting routine immunization services in the area. Fortunately, they were released through community-led negotiations involving the local partner WARDI. The disruption lasted for about a month before services resumed, and the team successfully reached zero dose children in the affected areas. The lesson through this incident includes trust building and adherence to the mutual agreements with elders and acceptance from the community are critical to ensure project implementation safely and successfully. It also posed importance to focus on recruiting individuals who can navigate local dangers, effectively communicate, and build relationships with the communities we serve.

We also explored the challenges families face. Through informal discussions with community members and parents/caregivers on why children are not vaccinated, we observed three recurrent themes: (i) lack of health facilities, (ii) safety concerns and (iii) opportunity cost.

## Sustainability potential

The predominant health service providers within project’s target areas are private pharmacies; therefore, the project emphasized on public–private partnership through capacity building, installing solar powered fridges and replenishment of vaccines stocks to ensure immunization service delivery are free of charge from these private pharmacies.

State-level Ministries of Health (MOHs) also coordinate with donors and utilize local resources (e.g. tax revenue) to support health service delivery within project locations. The FARID project supported State MoH to identify existing health facilities to re-establish health systems in these locations and to train health personnel for effective primary health care service delivery after the project ends.

## Conclusion

The key areas from FARID project implementation appear as attributable to project’s success:

- Clan elders’ engagement was critical for project planning, implementation, and monitoring.
- Delivery of integrated primary health care package created demand for service and community trust.
- Having local community-based organizations as implementing partners was the key for reaching the inaccessible population.

The project follows three principles in project implementation: [1) Community participation, 2) Innovation in data and mapping for precision of locations, and 3) Integrated services responsive to community needs]; and accomplished successes towards achieving national health strategic plan milestones, including progress towards universal health coverage.

Additionally, the key lessons during the project implementation are enumerated below:

- Building strong relationships with local stakeholders and community leaders helps negotiate access to inaccessible areas and garner necessary support.
- Collaboration ensures comprehensive response to supply shortages or capacity gaps.
- Flexibility and adaptability to unforeseen events (e.g. natural disasters or security incidents) helps mitigate disruptions and maintain service delivery.
- Alternative recruitment strategies and leveraging existing expertise (e.g. by transferring qualified personnel from nearby areas) help overcome limited capacity.
